# Skeptical Optimism Scale (SkO): Initial Development and Validation

**DOI:** 10.3390/bs15081017

**Published:** 2025-07-27

**Authors:** Cătălina Oțoiu, Petru Lucian Curșeu, Lucia Rațiu

**Affiliations:** 1 Department of Psychology, Babeș-Bolyai University, 400015 Cluj-Napoca, Romania; petrucurseu@psychology.ro; 2 Department of Organization, Open Universiteit, 6419 AT Heerlen, The Netherlands

**Keywords:** skeptical optimism, creativity, grit, critical thinking disposition, validity, reliability

## Abstract

This study introduces the Skeptical Optimism Scale (SkO) and presents preliminary evidence of its content, construct, and criterion validity. Skeptical optimism refers to dispositional tendencies of having general positive expectations about the future, conditional on critical analysis and in-depth exploration of (potential negative) outcomes. We developed an initial pool of 31 items that explore positive expectations in three main life domains (finding solutions to difficult problems, mastering novel and challenging tasks, and effectively dealing with general life challenges) that were subject to content analysis by eight independent raters. The remaining items were tested for criterion and predictive validity in two samples (*N* = 198 and *N* = 417 participants). Factor analyses supported a three-factor structure and the refined 17-item version of the scale showed good reliability and validity. To support applications in settings requiring brief instruments, we also developed a 9-item version, preserving the factorial structure and psychometric qualities of the original scale. The results show that the 17 as well as 9-item SkO scales have a good criterion validity as they positively and significantly correlate with the core self-evaluation scale, critical thinking disposition, and grit. Moreover, our results show that the SkO has good predictive validity as it is the only significant predictor of the creativity quotient in our sample.

*“A skeptical optimist is very different from a cautious optimist. A cautious optimist wants to be successful but has fear that they will not or are not producing the desired result. A skeptical optimist uses their skepticism to systematically test novel ideas.”*

(Mather, 2008, p. 46)

## 1. Introduction

Creativity and innovation are buzzwords in the contemporary business environment. Organizations strive to become and remain innovative and aim to attract and maintain creative individuals. In this continuous effort to identify, hire, and promote creative individuals, various personality traits (and dimensions) were studied as correlates (predictors) of creativity (Eysenck, 1993; Wolfradt & Pretz, 2001). With respect to the big five dimensions, the literature to date supports the positive association between extraversion, openness to experience, and, to some extent, conscientiousness and creativity (Karwowski & Lebuda, 2016; Wolfradt & Pretz, 2001; Xu & Cheng, 2025). With respect to specific traits, intuition (Wolfradt & Pretz, 2001), creative self-efficacy (Haase et al., 2018), inquisitiveness (Sternberg, 1985), and optimism (Rego et al., 2012) are positively related to creativity, while psychoticism is negatively correlated with creativity (Eysenck, 1994). In their meta-analysis of creativity and personality, Feist (1998) states that “creative people are more autonomous, introverted, open to new experiences, norm-doubting, self-confident, self-accepting, driven, ambitious, dominant, hostile, and impulsive” (p. 299). At a glance, the profile of a highly creative individual contains a collection of some divergent traits. Creative performance seems to require confidence and optimism as well as critical analysis, caution, and skepticism at the same time. The popular literature has long acclaimed skeptical optimism as the “counterintuitive trait” that drives creative performance (Snow, 2014). To date, however, empirical evidence supporting this claim is scant. One reason is the lack of scales to evaluate this rather peculiar combination of personal attributes, such as confidence in own abilities and success (optimism) and doubtfulness and being difficult to convince (skepticism). We argue that this mix of attributes extensively acclaimed by the popular literature as driving creativity (Salzberg, 2011; Snow, 2014) actually captures the combination of optimism and avoidance motivation attributes which drive some of the key psychological processes underlying creative performance (Icekson et al., 2014). We build on the motivational (Icekson et al., 2014) and the individual differences (Peterson, 2000) and theoretical perspectives on optimism to argue that skeptical optimism is a key antecedent of creative performance. On the one hand, optimism directly predicts creativity due to the fact that it is linked to higher engagement in creative thinking and the generation of new ideas (Rego et al., 2012). Skepticism, on the other hand, has been shown to encourage a more analytical and cautious approach to analyzing ideas and situations by reducing susceptibility to misinformation and irrational beliefs (Pennycook, 2023). This leads to a more critical evaluation of potential outcomes, which is associated with a lower likelihood of engaging in risky behaviors (I. Smith & Giroux, 2019), and is in line with the avoidance aspect underlined in the approach-avoidance motivational theory of Icekson et al. (2014).

Previous research has theorized this odd association of optimism and avoidance motivation as a key predictor of creativity (Icekson et al., 2014). From a motivational standpoint, while optimism is typically associated with approach motivation (pursuit of positive outcomes through action), adaptive forms of optimism may also integrate avoidance motives characterized by a desire to prevent negative outcomes (Carver et al., 2000). Research on defensive optimism (Norem & Chang, 2002) describes adaptive optimism as a cognitive-affective stance in which individuals maintain positive expectations about the future while actively engaging in evaluative thinking and scenario planning in order to mitigate potential setbacks. Moreover, in their theoretical framework, Icekson et al. (2014) argue that the interaction between optimism and approach vs. avoidance motivation is key to understanding human creativity, as optimism is beneficial for creativity only when it is associated with avoidance motivation (a tendency to avoid possible threats and other negative outcomes). We build on the motivational arguments presented in the theoretical model of Icekson et al. (2014) of adaptive optimism as a blend of hopeful orientation towards the future and vigilant threat monitoring to argue that positive expectations about the future associated with careful analysis of outcomes and inquisitiveness are key antecedents of the cognitive, affective and volitional processes that spur creativity. Thus, in line with the motivational perspective on adaptive optimism, we argue that optimism contributes to creativity not simply by fostering confidence, positive affect, and commitment to action, but it enhances creative performance, especially when it is combined with avoidance motives that encourage in-depth exploration, adequate risk assessment, and perseverance in the face of potential failure. The literature on optimism as an individual difference also calls for further conceptual refinement to better capture and further investigate the potential benefits as a general human disposition towards entertaining a positive outlook on the self, reality, and others (Peterson, 2000). Although optimism has traditionally been celebrated as a beneficial trait, these calls for conceptual refinement have highlighted the need to distinguish naïve or unrealistic optimism from its realistic, adaptive form (Craig et al., 2021). Peterson (2000), in particular, stresses the importance of incorporating constructive skepticism (thinking patterns that doubt irrational or exaggerated claims and beliefs) into the conceptualization of adaptive optimism. Separately, both optimism and skepticism have their dark sides in relation to creativity and innovation. On the one hand, too much skepticism may lead to procrastination and can stifle creativity, while too much optimism can lead to unrealistic, sloppy, and superficial ideation that is ultimately detrimental to creativity. Moreover, optimism without scrutiny and inquisitiveness is delusion, while skepticism without hope risks descending into cynicism (Mather, 2008; Peterson, 2000).

We argue that the combination of these two traits creates a sweet spot for creative performance where systemic inquiry meets imaginative thinking (Mather, 2008). Individually, both traits have been previously discussed in relation to creativity (Mayer & Mussweiler, 2011; Rego et al., 2018; Rego et al., 2012), but the literature lacks an integrated perspective on these traits. This gap is due to the fact that studies have looked into these characteristics separately, and previous work on creativity shows the need to integrate a number of different personality traits, as creativity is not a function of a specific trait but rather dependent on a combination of different factors (Feist, 1998, 2010). The aim of this study is to bridge that gap by offering means to assess them, not as individual traits, but rather as an integrated trait one might possess, in line with the motivational (Icekson et al., 2014) and individual differences (Peterson, 2000) theoretical frameworks stated above. This also answers previous calls in the literature to clarify the nuances of the positive effect optimism has on creativity (Craig et al., 2021). We therefore set out to develop a scale that evaluates skeptical optimism, as a concept which encompasses aspects of optimism and skepticism in balance with each other, rather than separate traits, and to explore the content and criterion validity of this scale. This study enriches the creativity literature by rigorously examining the interplay between optimism and skepticism, showcasing how their complementarity fosters deeper cognitive processing and significantly enhances individual creative potential. Beyond offering a means to clarify skeptical optimism as a trait from a conceptual point of view, our study also provides an actual means to test it and therefore make practical decisions with regard to evaluating individual creative potential across organizations, educational environments, and developmental programs. Furthermore, the newly developed scale serves as a crucial tool for future research, especially in the realms of creativity and creative performance. We start by presenting the process of item development and the content validity of the scale and then we present the results of two empirical studies that investigate the construct validity (Study 1) and criterion validity (Study 2) of the scale.

## 2. Method

### 2.1. Scale Development

Optimism refers to the general expectations of positive outcomes in the future (Carver & Scheier, 2014; Schweizer & Koch, 2001). Previous research has documented the idiosyncratic and context-dependent nature of optimism (Palacios-Delgado & Acevedo-Ibarra, 2023) and called for conceptual refinements to capture its specific association with various adaptive outcomes (Peterson, 2000). As optimism is a dispositional tendency to use positive evaluative cognitions in relation to future events, any evaluation instrument should include items referring to several life domains in order to capture this generic tendency across them. Building on the theoretical arguments grounding the development of the self-efficacy optimism scale (Schweizer & Koch, 2001), we have identified three core domains in relation to which skeptical optimism should be defined, namely (1) finding solutions to difficult problems, (2) master novel and challenging tasks, and (3) effectively deal with general life challenges. Skeptical optimism reflects a tendency to have general positive expectations about the future, conditional on inquisitiveness and curiosity, critical analysis, and in-depth exploration of plausible (negative) outcomes. Several validated scales that evaluate dispositional optimism are already in use, and we started the item development from these existing instruments (Coelho et al., 2018). However, it is our intention to capture a particular set of individual attributes that also include, next to the positive expectations for the future (in various life domains), the conditional effect of inquisitiveness, careful scrutiny, and persistence in overcoming difficulties. Therefore, the general strategy to formulate items was to phrase the positive (optimistic) expectations about the future conditional on persistent scrutiny and critical evaluation of the (negative) outcomes across the three core domains mentioned earlier (i.e., “For each problem I am confronted with I will find a solution because I tend to think a lot about things”).

For the development of the initial item pool for this scale, three experts in Applied Social Psychology generated several items in the three life domains mentioned above. In total, 31 items were generated, and the content of these items is presented in Appendix A. We have distributed this initial pool of items to a sample of students, and asked them to also perform an idea generation task (namely generate as many uses as possible for an apple) for the purpose of obtaining a measure of creativity by computing the creativity quotient introduced by Snyder et al. (2004). In order to test for concurrent validity, we also added the following scales to this data collection phase, namely core self-evaluations (Judge et al., 2003), grit (Duckworth & Quinn, 2009), critical thinking disposition (Sosu, 2013), and self-efficacy optimism (Schweizer & Koch, 2001). In order to check whether the items consistently cover the three intended domains, we have asked a number of eight independent raters to evaluate the phrasing of the items and to allocate them in their respective categories, according to their content to the three life domains. In order to check the agreement across the experts we have computed the within-group agreement score RWG (James et al., 1993) across the eight evaluators and we used values higher than 0.60 as a cutoff point, under the assumption that higher RWG values reflect a higher level of agreement or the true score consensus across the raters (Newman & Sin, 2020). The content of the initial item pool and the results of the RWG scores as well as the results of the initial Exploratory Factor Analysis and correlation with other criterion variables are presented in Appendix A.

### 2.2. Sample and Procedure for Study 1 and Study 2

For the initial test of item validity (Study 1), we have used a student sample (*N* = 198, 163 women, age *M* = 21.75, *SD* = 1.39). An additional sample of 417 participants was used to further test the validity of the scale (Study 2—*N* = 417, 300 women, age *M* = 23.72, *SD* = 6.43). We have used a convenience sampling method for our first study, with participants being enrolled in an organizational psychology class for their BA program at our university. For our second study, we used a snowball sampling method starting with a different convenience sample made of students enrolled in an organizational psychology class for their BA program in our university, and asking them to collect data from their individual networks. This was relevant because of the fact that our second measure of creativity involved addressing creative accomplishments across various life domains, and hence, a larger and more heterogeneous sample was required. The students participated in both studies in exchange for research credits, and they had to fill in an informed consent form. The participant students recruited in the second study also had to fill in the informed consent form.

### 2.3. Measures for Study 1 and Study 2

The Skeptical Optimism Scale included 31 items generated in the initial stage by three experts, across three domains: finding solutions to difficult problems (SDP “For each problem I am confronted with I will find a solution because I tend to think a lot about things.”), mastering novel challenging tasks (NCT “Curiosity makes me able to fulfill new and challenging tasks”), and dealing with general life challenges (GLC “In my life, I tend to question even most basic assumptions”). In order to check whether the items clearly reflect these distinct domains, we asked eight raters to distribute the 31 items across the three core domains, with the intention of maintaining the items that the raters generally agreed belonged to a particular domain. Answers were recorded on a 5-point Likert scale ranging from 1 = fully disagree to 5 = fully agree.

#### 2.3.1. Study 1

Creativity was first assessed using a creativity quotient, a divergent thinking metric introduced by Snyder et al. (2004). Participants had to complete an idea generation task in which they were invited to generate as many uses as possible for an apple. The ideas generated by the participants were coded by two raters in 14 distinct categories (i.e., eat raw, eat processed, sports and games, decoration, proverbs, stories, etc.) based on an initial screening of the pool of ideas generated. The categories were derived by progressively scanning the ideas and adding new categories when new ideas appeared in the lists. A retrospective coding was carried out after each new category was added to the set, so that all generated lists were coded using the same categories. The creativity quotient was then computed using a procedure described in (Lucas et al., 2013) that includes the fluency (number of ideas generated), flexibility (the number of categories in which the ideas can be organized), and the diversity of the ideas generated by the participants. The intraclass correlation coefficient for the two creativity quotient scores derived from the expert coding was 0.92, showing substantial consistency in the two ratings. As a consequence, we averaged the two scores into a generic creativity quotient to be used as a criterion variable in the analyses.

Grit was assessed with the short grit scale (Grit-S) developed by Duckworth and Quinn (2009). This version of the scale includes 8 items which reflect the two dimensions of grit—consistency of interest (i.e., “My interests change from year to year.”) and perseverance of effort (i.e., “I finish whatever I begin.”). Items were rated on a 5-point Likert scale with options ranging from 1 = completely disagree to 5 = completely agree. The Cronbach’s alpha of this scale was 0.81.

Core self-evaluation was evaluated with the 12-item scale developed and validated by Judge et al. (2003). The scale measures positive feelings about the self in terms of self-esteem, generalized self-efficacy, emotional stability, and locus of control. Participants used a 5-point scale with response options ranging from 1 = strongly disagree to 5 = strongly agree to rate themselves on the items. A sample item is “Overall, I am satisfied with myself”. The Cronbach’s alpha of this scale was 0.82.

Self-efficacy optimism was assessed using the self-efficacy optimism subscale in the POSO-E (Schweizer & Koch, 2001), a measure that assesses three components of optimism: personal, social, and self-efficacy optimism. The subscale we used for self-efficacy optimism comprises 10 items, statements expressing the expectation of a positive consequence of one’s own behavior. All items were rated on a four-point scale with the response options being “completely correct”, “almost correct”, “partly correct”, and “incorrect”. A sample item is “I always find a solution to a problem”. The Cronbach’s alpha for this scale was 0.88.

Critical thinking disposition was assessed with the critical thinking disposition scale (CTDS) developed by Sosu (2013). The measure includes 11 items rated on a five-point Likert scale where 1 = strongly disagree and 5 = strongly agree. Seven of the items reflect a disposition towards critical openness (i.e., “I sometimes find a good argument that challenges some of my firmly held beliefs”) and four items indicate a disposition towards reflective skepticism (i.e., “I often re-evaluate my experiences so that I can learn from them”). The Cronbach’s alpha for this scale was 0.74.

#### 2.3.2. Study 2

In order to test for the predictive validity of the Skeptical Optimism Scale, in Study 2, we evaluated creativity with the Creative Achievement Questionnaire (CAQ) (Carson et al., 2005), a self-report measure which uses 96 items to assess achievements across 10 domains of creativity (visual arts, music, dance, creative writing, architectural design, humor, theater and film, culinary arts, inventions, and scientific inquiry). Participants were asked to place a checkmark next to the areas in which they perceive they have higher than average talent or ability. Each domain consisted of eight ranked statements (weighted with a score ranging from 0 to 7) on which respondents had to indicate whether the statement described their accomplishments (yes = 1, no = 0). The Cronbach’s alpha of this scale was 0.96.

## 3. Results

### 3.1. Factorial Analyses

Because the content of the items was newly generated, we first performed an Exploratory Factor Analysis (EFA) with an Oblimin rotation using the JASP software version 0.19.3.0, for the 31 items to check the extent to which items tend to cluster in specific factors. We combined the emergent factors from the EFA with the rating agreements and maintained the items for which the RWG score (as an indicator of true score consensus among raters) was higher than 0.60 and the items loaded in specific factors as illustrated by factor loadings higher than 0.40. The specific EFA factor loadings for Study 1 are presented in Appendix A. We then correlated the 31 items with the scores for the scales used as criteria for the validation process. These correlations are also reported in Appendix A.

We triangulated these analyses in order to select the most valid indicators of skeptical optimism such that we selected (1) items on which the experts had a generic agreement when asked to distribute them to their respective dimension, (2) items that had factor loadings higher than 0.40 in the EFA, and (3) items that displayed significant correlations with (some of) the criterion scales. Three factors emerged from this selection process, which ultimately included 17 items clustered in three factors aligned with the intended constructs: (1) factor 1—curiosity in mastering novel and challenging tasks, (2) factor 2—tenacity in scrutinizing difficult problems, and (3) factor 3—inquisitiveness in addressing challenges. The content of items and their respective factor loadings as derived from CFA. We conducted CFA analysis with Maximum Likelihood Estimation and robust standard errors using JASP software version 0.19.3.0 (when using the MLR and DWLS estimators, the results remained unchanged). The CFAs separately for the samples from Study 1 and Study 2 are presented in Table 1. We have also replicated the CFA using AMOS version 23, and the results were similar to the ones reported in Table 1.

These selected items were used for the additional analyses. We first carried out additional CFA analyses and tested different models, namely the three-factor model, the three-factor model with an underlying single latent dominant factor, and the single-factor model. The results of the CFA are presented in Table 2.

As indicated in Table 2, the best fitting model is the three-factor model with covariates. The Cronbach’s alpha for the overall questionnaire as well as for the separate scales are all above 0.70, indicating good internal reliability (see Table 3 for scores). We also estimated the omega score based on a confirmatory factor analysis procedure (Hayes & Coutts, 2020), and the values also indicate a good reliability of the overall skeptical optimism questionnaire as well as for its separate scales. Cronbach’s alpha and omega scores are presented in Table 3. The square root value of the average variance extracted for each of the three factors (AVE) was higher than the interfactor correlations, supporting the discriminant validity of the three factors of the SkO scale (Fornell & Larcker, 1981). Overall, the AVE scores are above 0.43, showing that the underlying factors explain at least 43% of the variance of the indicators.

Our main aim was to develop a valid and reliable measure of skeptical optimism that can be used in research and practice. The 17-item measure holds its factorial structure in the two studies, and the subscales assess distinct dimensions of skeptical optimism. We are aware that in larger research projects in which indicators of skeptical optimism are deemed relevant, researchers may face the limitations associated with large surveys that reduce responsivity, and as such, they may look for more parsimonious ways of assessing skeptical optimism. We therefore present below additional analyses for a shorter version of the scale, including only three items per factor.

#### A Short Version of the Skeptical Optimism Scale (SkO-Short)

In developing a shorter version of the scale, we aimed to maintain the factorial integrity across the three factors and maintain acceptable levels of internal consistency for the subscales. We therefore decided to select three items from each subscale, based on their factorial loading, in such a way that engaging with difficult problems, new challenging tasks, and life challenges are represented across the scales. The short version of the skeptical optimism therefore includes nine items, three for each of the three factors: curiosity in mastering novel and challenging tasks, tenacity in scrutinizing difficult problems, and inquisitiveness in addressing challenges. The selected items for the short version of the scale are presented in bold in Table 1. Similar to the initial scale, we carried out CFA on the Study 1 and Study 2 samples. The results of the CFA with three correlated factors are presented in Table 4, while Cronbach’s alpha and omega scores for the subscales and the overall short scale are presented in Table 5.

The results of the CFA for the short scale are similar to the results for the initial scale, and the internal reliability of the subscales is maintained. Moreover, the square root of the AVE scores was higher than the interfactor correlations, supporting the discriminant validity of the three factors of the SkO scale. In terms of convergent validity, the AVE scores for the short version of the SkO scale are higher than for the 17-item version of the scale, showing that for the short scale, the underlying factors explain a higher percentage of the variance in the indicators (Fornell & Larcker, 1981). All in all, the CFA analyses show stronger support for the shorter version of the scale. Further on, for the validation studies, we will proceed with both scores for the 17- as well as the 9-item version of the Skeptical Optimism Scale.

### 3.2. Criterion Validation

The correlations between the three subscales of skeptical optimism and the criterion variables are presented in Table 6.

In order to further check the predictive power of each of the subscales, we ran OLS regression analyses with the criterion variables and the subscales of skeptical optimism as predictors. The results are presented in Table 7 and Table 8.

Overall, the correlations between the criterion variables and the dimensions of SkO, as well as for the overall score of the short scale, were positive and significant, supporting the validity of the scale.

### 3.3. Predictive Validity

The correlation between the SkO subscales (as well as the total score for the scale) and the Creative Achievement Questionnaire is presented in Table 9.

As indicated in Table 9, all subscales of the SkO scale correlate positively and significantly with the Creative Achievements Questionnaire, and the same is valid for the short version of the subscales. These results further support the predictive validity of the SkO scale.

## 4. Discussion

The aim of our research was to develop and to validate a measure for the assessment of skeptical optimism as an adaptive form of optimism that spurs creative performance. We defined skeptical optimism as a combination of healthy skepticism and optimism reflected by a simultaneous focus on commitment to action in the hope of achieving positive outcomes with careful scrutiny and assessment of potential risks, and we argued that it encourages in-depth information processing and ultimately fosters creativity. Based on validation tests carried out in two studies, a 17-item scale was developed, as well as a shorter 9-item version from the original pool of 31 items generated by experts. Exploratory factor analyses and subsequent confirmatory factor analyses on two different samples yielded a three-factor structure of our scale, with the three subscales being curiosity in mastering novel and challenging tasks, tenacity in scrutinizing difficult problems, and inquisitiveness in addressing challenges. Scale reliability was high for both versions of the instrument.

In developing this measure of skeptical optimism, we intended to contribute to the advancement of research on creativity by offering a tool that would be able to assess a concept, which was promoted as one of the driving forces behind creative behaviors (Snow, 2014). In this following section, we discuss our findings on the nature of skeptical optimism from a conceptual standpoint, linking it to the dual regulatory focus (Higgins, 1997) and the approach-avoidance motivational approach to creativity (Icekson et al., 2014), where we grounded our theoretical arguments. We then showcase its relation to creativity and creative performance and explain how our measure, as a whole, as well as each of its three subscales, highlights and contributes to uncovering this relation. We then proceed to discuss the limitations of our work, potential future research directions and, finally, the implications of this study.

### 4.1. Skeptical Optimism as a Dual-Focus Disposition Driving Creativity

Our findings underscore skeptical optimism as an integrative trait that combines a dual regulatory focus on promotion and prevention (Higgins, 1997), and it is aligned with Feist’s (2010) claim that creative performance reflects a complex and sometimes contradictory blend of traits such as skepticism and optimism. While these two traits may seem contradictory, their co-existence may reflect a high degree of meta-cognitive sophistication, allowing individuals to maintain hope while also being inquisitive in relation to the world around them. This conceptualization aligns with motivational frameworks that integrate approach and avoidance motivations with optimism (Carver & Scheier, 2014) and supports the dual process nature of skeptical optimism, as derived from the model presented in Icekson et al. (2014), such that an optimistic outlook drives perseverance in achieving goals while skepticism promotes in-depth exploration. In their updated model on creative thought or behavior, Feist (2010) expands their initial argument on the link between personality and creativity by looking into the genetic and epigenetic influences on four categories of personality traits, which in turn influence creative behaviors. Cognitive personality traits (i.e., openness to experience) explain how people habitually process information and deal with new situations. Social personality traits (i.e., norm-doubting, nonconformity, and independence) describe how one builds relationships with other people, especially with regard to reactions to authority figures and general social norms. Motivational-affective personality traits (i.e., perseverance, impulsiveness) are defined as a person’s desire to be effective and persist in their tasks. Lastly, certain clinical personality traits (i.e., psychoticism, latent inhibition) have been linked to creativity through mechanisms such as holistic integration of ideas rather than in a strict and analytical manner. Feist (2010) proposes that these four dimensions of personality build on each other in order to influence behavior. Amănălăchioaie et al. (2025) show, for example, how people with dispositional optimism, through their positive evaluations of their social environment (cognitive traits) and their tendency to pursue and exert effort towards social goals (motivational-affective dimension), also tend to have a richer social network structure. Through similar reasoning, Feist (2010) states that it is a combination of these counterintuitive personality traits that lowers behavioral thresholds, making creative thought and behaviors more likely (Feist, 1998, 2010).

Our results are generally aligned with these arguments, showing that all three factors of the SkO scale correlate positively with the creativity quotient evaluated in the idea generation task (see Table 6). However, when all three factors are considered multivariate predictors of the creativity quotient, only factor 1 (curiosity in mastering novel and challenging tasks) positively and significantly predicted creativity. The second (tenacity in scrutinizing difficult problems) and third (inquisitiveness in addressing challenges) factors showed comparatively lower positive association with the creativity quotient (see Table 7 and Table 8). These differential associations are theoretically consistent with the differentiated cognitive pathways activated by these traits. While curiosity in mastering novel and challenging tasks is more directly associated with divergent thinking, as it fosters cognitive fluency and idea generation, the other two factors capture deeper and more reflective information processing pathways such as task persistence and inquisitiveness. As tenacity in scrutinizing difficult problems and inquisitiveness in addressing challenges are essential for creative processes, especially in later stages that include implementation and refinement of creative ideas, they are less likely to enhance spontaneous idea generation and ideational fluency as captured by the creative quotient used in our first study. Future research using more refined measures of various creative processes may better capture the relationship between the three factors of the SkO and creativity. In particular, tenacity and inquisitiveness may not drive ideational fluency, yet they can sustain problem engagement, help reframing ideas, as well as idea testing and implementation, all key aspects of the creative processes. We call for more research building on dual pathways to creativity (Cropley, 2015; Nijstad et al., 2010) to further explore the association between the SkO scale and idea generation as well as the secondary processes associated with problem engagement, critical reflection, and deep evaluative judgments. Moreover, exceptional creative achievements in science, art, and engineering often require long-term commitment and intellectual engagement (Amabile, 2018); therefore, such extreme forms of creative achievement could be fostered by tenacity and inquisitiveness. Taken together, these findings support the view that factors 2 and 3 of the SkO scale contribute more subtly to creative performance by facilitating idea refinement and evaluation as well as in-depth information processing that facilitates effective problem solving. While these two factors may not significantly predict creativity as assessed by traditional divergent thinking measures, they are more likely to play an important role in the later-stage creative processes and innovation, such as evaluation, mobilizing support, and implementation of creative ideas.

As such, skeptical optimism reflects a dispositional tendency to expect positive results for the future, contingent on critical analysis and in-depth exploration of their (potential negative) outcomes. Skeptical optimists, therefore, balance an optimistic approach to dealing with new or challenging tasks and situations with an inclination towards skeptical inquiry. Both skepticism and optimism, on their own, have a positive impact on creativity, but could also generate potentially negative effects. Hence, it is specifically this balance that has been previously proven to play a crucial role in creative performance (Rego et al., 2018).

Optimism fosters positive affect, confidence, and persistence and, through these mechanisms, has a positive effect on creativity and creative problem solving in complex task situations (Williams, 2014). Moreover, optimistic beliefs seem to enhance creative self-efficacy, which, in turn, boosts creativity (Zhang et al., 2019). Excessive optimism, however, can be detrimental because it generates complacent behaviors and reduces the critical evaluation of situations (Rego et al., 2018).

Skepticism can enhance creativity by promoting cognitive flexibility. Because of their tendency to distrust and question new information or situations, skeptical individuals are more likely to consider non-obvious alternatives and think outside the box (Mayer & Mussweiler, 2011). However, the social consequences of skepticism, such as reluctance to share information, can sometimes hinder creativity, especially in collaborative settings (Mayer & Mussweiler, 2011).

The interplay between optimism and skepticism is therefore crucial. While optimism can reduce anxiety and threat appraisals and maintain a positive outlook on a situation, skepticism can prevent complacency and encourage critical thinking, leading to more innovative solutions (Icekson et al., 2014; Mayer & Mussweiler, 2011). Our measure of skeptical optimism reflects exactly this interplay. The three-factor structure of the scale targets crucial aspects related to optimism (curiosity, positive outlook, persistence, and hope) and complements them with the inquisitiveness and critical thinking disposition aspects of skepticism.

### 4.2. Curiosity in Mastering Novel and Challenging Tasks

Positive emotions associated with optimism can enhance cognitive flexibility, which is crucial for generating novel ideas (Rego et al., 2012). Optimism may also trigger inhibitory control processes, which help in resisting spontaneous, less creative solutions and exploring more original ideas (Cassotti et al., 2016). Through different processes, like distrust in early solutions and the inclination to look for more options and to critically analyze them, skepticism also supports cognitive flexibility and the generation of alternative ways to deal with challenges.

In their development of a critical thinking disposition scale, Sosu (2013) draws on the existent taxonomies on important thinking dispositions and combines aspects such as inquisitiveness, trust in reasoned inquiry, flexibility in considering alternatives, prudence in making judgments, tolerance for ambiguity (Facione & Facione, 1992; Halonen, 1995), open-mindedness, adventurousness, and sustaining intellectual curiosity (Facione & Facione, 1992; Perkins et al., 1993). They define their two facets of critical thinking disposition as reflective skepticism and critical openness (Sosu, 2013). Our results show that our subscale of *curiosity in mastering novel and challenging tasks* has positive and significant correlations with both subscales of critical thinking disposition demonstrating an effective grasp of both aspects. Moreover, this finding underlines the need to combine inquisitiveness and critical analysis with innate curiosity and the open-mindedness needed for creative thinking.

### 4.3. Tenacity in Scrutinizing Difficult Problems

Optimism is a significant predictor of grit, defined as the perseverance and passion for long-term goals (Duckworth et al., 2007). Due to higher levels of grit and their tendency to expect positive outcomes, when faced with difficult situations, optimistic individuals are more likely to persevere through challenges (Pyo et al., 2024; Wong et al., 2024). Especially within learning environments, optimism provides the necessary resources for gritty people to achieve better outcomes and higher performance (Luthans et al., 2019). Optimism associated with a growth mindset has a positive impact on the perseverance of the effort component of grit (Pyo et al., 2024), and optimistic individuals with high grit are more likely to experience a state of deep engagement and flow in their activities (Kim & Lee, 2021).

In our study, we found a significant and positive correlation between our *tenacity in scrutinizing difficult problems* subscale and grit, both in terms of perseverance and consistency of effort.

### 4.4. Inquisitiveness in Addressing Challenges

This third subscale of our instrument captures more directly the impact that skepticism brings to creative performance. It reflects on how people tend to make decisions with regard to life challenges and new situations, based on a disposition to view them in a critical manner and to systematically try and find alternative or innovative ways of dealing with them, as opposed to walking the beaten path. Such detailed scrutiny in information processing was supported by the positive association between the inquisitiveness subscales and both dimensions of the critical thinking disposition scale. When faced with a difficult new situation or challenge, skeptical individuals tend to employ critical thinking and rational judgment rather than rely on intuitive thinking (Zhang et al., 2023). They tend to question the feasibility of solutions, especially when there is a perceived imbalance between the perceived complexity of the situation and the readily proposed solutions (Trieste & Turchetti, 2024). Being open to addressing novel ideas is balanced by the need to ensure that there is sufficient domain expertise to assess their feasibility and not take them at face value but to critically evaluate them (Rietzschel et al., 2023). Inquisitiveness, however, has its downsides, as illustrated by its negative association with both facets of GRIT and CSE. When the other two dimensions of skeptical optimism are controlled, the remaining association of inquisitiveness with grit and CSE was negative, pointing out the daunting effect inquisitiveness has on motivational perseverance.

In sum, skeptical optimism provides a valuable lens to understand creative performance. By integrating hopeful engagement with rigorous scrutiny, individuals scoring high on SkO are better equipped to navigate the uncertainties of creative tasks and are more effective in imagining new possibilities, while remaining grounded in critical evaluation.

### 4.5. Limitations and Future Research Directions

Despite the robust psychometric properties of the SkO scale, several limitations should be acknowledged. First, both validation studies relied solely on self-report data for the criterion variables, raising concerns with the common method bias, especially in the second study in which creative achievement was self-rated. The subscales correlated significantly with the creative achievement score, yet the percentage of variance explained in the creativity quotient was rather low. Future research could expand these results and correlate SkO with creative achievements throughout life as rated by external knowledgeable raters. In Study 1, we have used a well-established metric for creativity and an external rater coded individual creative performance, yet the other criterion variables included in Study 1 were self-reported. Second, our studies were cross-sectional, and we cannot draw any causal claims based on our results. Future studies could attempt manipulations of skeptical optimism (as a state) and explore its causal role more precisely. Also, future studies could further test the psychometric qualities of the SkO scale in larger samples, and also, using longitudinal designs, future research could explore the stability of skeptical optimism over time. The SkO integrates dual motivational processes, and because it captures an apparent paradox of integrating a promotion and a prevention focus, paradox theory (W. K. Smith & Lewis, 2011) could be used to further explore this construct. In particular, we see similarities with the construct of integrative complexity (Tetlock, 1985) that effectively combines cognitive differentiation and integration in complex cognitive structures (Tucaliuc et al., 2024). Future studies could explore the association between integrative complexity and skeptical optimism, as both constructs reflect a paradoxical combination of apparently opposing processes and mechanisms. While our study explored the association between skeptical optimism and individual creativity, its role in group or collaborative creativity is also worthy of exploration. Group members exhibiting varying levels of skeptical optimism engage with the collaborative group processes differently, ultimately shaping the creative and innovative outcomes of groups (Pluut & Curșeu, 2013). Alternatively, we could envisage that a single group member scoring very high on skeptical optimism could engage more actively in minority dissent, steering the collaborative group processes (Curșeu et al., 2022). By extending these research avenues to explore collaborative creativity, scholars could develop multilevel theoretical frameworks that further nuance the relationship between skeptical optimism and creative performance of individuals, groups, and organizations. Furthermore, as the inquisitiveness in addressing challenges is a key component of SkO, future research could also explore the role of skeptical optimism in driving attitudes and engagement with actions aligned with the Sustainable Development Goals aimed at addressing some of the most important modern societal challenges and transitions. Finally, future research could also explore cross-cultural differences in skeptical optimism as it relates to creative achievements in various settings and cultures.

### 4.6. Practical Implications

Beyond the theoretical contributions to the further exploration of optimism and its facets, revealing how the synergistic interplay between optimism and skepticism fuels deeper cognitive processing and enhances the creative potential, the newly developed SkO scale also has important practical implications. Organizations could use the scale in training and hiring processes in order to identify individuals who can perform effectively in creative tasks or complex problem-solving situations where a critical balance between hope and caution is paramount. In educational settings, the SkO can be integrated into interventions aimed at further developing critical thinking in students. Educators could use the scale to track the evolution of students’ cognitive dispositions and tailor their teaching methods to foster persistence and inquisitiveness. For students who aim to pursue a career in creative industries, the SkO can be a useful reflective tool to help them harness their creative potential to the fullest. In coaching and personal development frameworks, the SkO scale could help professionals harness their optimistic drive while reinforcing inquisitive thinking habits. Given the synergistic combination of hope and doubt captured by the SkO scale, managers could also use it to make team allocation decisions, as the scale offers critical insights into individual motivational profiles and the way in which they can also contribute to boost the creative potential of teams.

## 5. Conclusions

This study presents the development and validation of the Skeptical Optimism Scale (SkO) as an option for assessing the “counterintuitive trait”—a combination of optimism and skepticism integrating dual motivational processes focused on prevention and promotion, with positive impact on human creativity.

The SkO scale showed strong psychometric properties with a consistent three-factor solution, capturing distinct but related domains: curiosity in mastering novel and challenging tasks, tenacity in scrutinizing difficult problems, and inquisitiveness in addressing challenges. We found positive correlations between skeptical optimism and grit, self-efficacy optimism, core self-evaluation, and critical thinking disposition, which underline this dual and almost contradictory nature of skeptical optimism. Moreover, our scale has good predictive validity indicators with respect to creativity and creative achievement. The Skeptical Optimism Scale (SkO) provides a valuable tool for future research, especially in the study of creativity and creative performance.

## Figures and Tables

**Table 1:** Factorial structure and CFA factor loadings.

Factor	CFA FL Study 1		CFA FL Study 2		Item
	Ustd.	Std.	Ustd.	Std.	
Factor 1 (curiosity in mastering novel and challenging tasks)	1.000	0.703	1.000	0.712	Curiosity makes me able to fulfill new and challenging tasks
	1.163	0.734	1.133	0.700	I am excited when faced with new challenges.
	**0.806**	**0.619**	**1.106**	**0.728**	I enjoy complexity in life due to my curiosity *
	**1.187**	**0.843**	**1.251**	**0.815**	**I like exploring new and challenging tasks**
	**1.058**	**0.746**	**1.170**	**0.772**	**I like experimenting because it challenges me to think quickly and flexibly**
	0.782	0.547	0.862	0.559	I like to think out of the box when faced with difficult/new problems.
	1.118	0.754	1.232	0.781	I welcome every new challenge because it triggers my inquisitiveness
	0.945	0.644	1.232	0.822	My curiosity helps me deal with new and challenging tasks
Factor 2 (tenacity in scrutinizing difficult problems)	1.000	0.485	1.000	0.524	My persistence helps me deal with any difficult life situation
	**0.993**	**0.587**	**0.959**	**0.523**	**For each problem I am confronted with I will find a solution because I tend to think a lot about things.**
	**1.784**	**0.772**	**1.660**	**0.787**	**I am willing to engage and persist in a complex task because I always find a way to get it done.**
	**1.466**	**0.695**	**1.571**	**0.700**	**I believe I will always find a way when dealing with difficult challenges.**
	1.264	0.631	1.557	0.739	I have the drive to complete novel tasks no matter their complexity
Factor 3 (inquisitiveness in addressing challenges)	1.000	0.481	1.000	0.391	I often wonder about why something works or doesn’t work.
	**1.402**	**0.551**	**1.462**	**0.427**	**In my life, I tend to question even most basic assumptions**
	**1.588**	**0.816**	**2.523**	**0.861**	**I sometimes overanalyze a problem because I want to make sure I reach the best solution.**
	**1.568**	**0.804**	**2.376**	**0.850**	**I sometimes overanalyze a new situation because I want to make sure I understand all aspects of it.**

Notes: all factor loadings are significant at *p* < 0.001; Std. = standardized factor loading; Ustd. = unstandardized factor loading; CFA FL—factor loadings derived from the confirmatory factor analysis; * items in bold are included in the short skeptical optimism scale.

**Table 2:** Results of the confirmatory factor analysis for studies 1 and 2—17 items.

Model	χ^2^ (*p*)	Df	CMIN/Df	RMSEA	CFI	TLI	NFI
*Study 1*							
3 factor model	245.18 (<0.0001)	116	2.11	0.08	0.90	0.89	0.89
3 factor model (with an underlying dominant factor)	481.93 (<0.0001)	119	4.05	0.13	0.73	0.69	0.67
Single factor model	484.39 (<0.0001)	119	4.07	0.13	0.73	0.69	0.69
*Study 2*							
3 factor model	392.63 (<0.001)	116	3.38	0.08	0.92	0.90	0.90
3 factor model (with an underlying dominant factor)	882.96 (<0.001)	119	7.42	0.12	0.77	0.74	0.74
Single factor model	885.08 (<0.001)	119	7.44	0.12	0.77	0.74	0.74

Note: the three factors were allowed to covary, and the best fitting models are the three-factor model with covariates between the factors.

**Table 3:** Reliability and average variance extracted for the scales in Study 1 and Study 2 (17-item version).

	Study 1			Study 2		
	Coefficient ω	Coefficient α	AVE	Coefficient ω	Coefficient α	AVE
Factor 1 (curiosity in mastering novel and challenging tasks)	0.884	0.884	0.50	0.906	0.903	0.55
Factor 2 (tenacity in scrutinizing difficult problems)	0.777	0.770	0.43	0.792	0.795	0.46
Factor 3 (inquisitiveness in addressing challenges)	0.728	0.755	0.43	0.707	0.731	0.56
Total	0.869	0.887		0.882	0.894	

Note: AVE—average variance extracted.

**Table 4:** Results of the confirmatory factor analysis for the short version of the scale (9 items).

Model	χ^2^ (*p*)	Df	CMIN/Df	RMSEA	CFI	TLI	NFI
Study 1–3 factor model	52.12 (<0.0001)	24	2.17	0.07	0.95	0.93	0.93
Study 2–3 factor model	84.63 (<0.0001)	24	3.52	0.08	0.96	0.94	0.94

Note: the three factors were allowed to covary.

**Table 5:** Reliability and average variance extracted for the short scale version (9 items) in Study 1 and Study 2.

	Study 1			Study 2		
	Coefficient ω	Coefficient α	AVE	Coefficient ω	Coefficient α	AVE
Factor 1 (curiosity in mastering novel and challenging tasks)	0.785	0.767	0.56	0.821	0.818	0.61
Factor 2 (tenacity in scrutinizing difficult problems)	0.752	0.735	0.51	0.720	0.719	0.48
Factor 3 (inquisitiveness in addressing challenges)	0.741	0.729	0.49	0.745	0.718	0.50
Total	0.809	0.808		0.842	0.788	

**Table 6:** Means, standard deviation, and correlations for variables included in Study 1.

	Mean	SD	1	2	3	4	5	6	7	8	9	10	11	12	13	14	15
1. Gender	0.83	0.379	1														
2. Age	21.75	1.395	−0.138	1													
3. Factor_1	3.68	0.688	−0.115	0.013	1												
4. Factor_2	3.81	0.638	−0.044	0.004	0.570 **	1											
5. Factor_3	3.92	0.710	−0.010	−0.053	0.388 **	0.317 **	1										
6. SkO_Tot	3.76	0.537	−0.070	−0.019	0.890 **	0.803 **	0.608 **	1									
7. Factor_1SH	3.77	0.735	−0.092	0.000	0.943 **	0.542 **	0.385 **	0.847 **	1								
8. Factor_2SH	3.88	0.718	−0.054	−0.011	0.562 **	0.913 **	0.327 **	0.765 **	0.532 **	1							
9. Factor_3SH	3.88	0.760	0.004	−0.083	0.352 **	0.309 **	0.961 **	0.573 **	0.341 **	0.318 **	1						
10. SkO_TotSH	3.84	0.570	−0.060	−0.041	0.798 **	0.754 **	0.730 **	0.940 **	0.805 **	0.790 **	0.725 **	1					
11. Q_Creat	6.94	2.604	−0.012	0.048	0.291 **	0.186 **	0.192 **	0.297 **	0.288 **	0.203 **	0.170 *	0.284 **	1				
12. CSE	3.57	0.589	−0.030	0.026	0.203 **	0.339 **	−0.256 **	0.204 **	0.155 *	0.292 **	−0.272 **	0.068	0.025	1			
13. GRITpersev	3.62	0.757	0.114	−0.021	0.224 **	0.459 **	−0.089	0.307 **	0.161 *	0.308 **	−0.087	0.160 *	−0.121	0.594 **	1		
14. GRITconsist	3.00	0.966	0.196 **	0.034	−0.006	0.270 **	−0.201 **	0.077	−0.048	0.227 **	−0.190 **	−0.010	−0.049	0.426 **	0.436 **	1	
15. CTD_CO	3.95	0.498	−0.005	0.034	0.478 **	0.360 **	0.496 **	0.544 **	0.444 **	0.350 **	0.461 **	0.542 **	0.163 *	0.117	0.105	−0.060	1
16. CTD_RO	4.11	0.567	0.196 **	−0.005	0.329 **	0.355 **	0.341 **	0.424 **	0.303 **	0.316 **	0.332 **	0.410 **	0.030	0.222 **	0.364 **	0.164 *	0.506 **
17. SEOpt	3.56	0.607	−0.079	0.040	0.448 **	0.637 **	0.126	0.554 **	0.402 **	0.569 **	0.126	0.467 **	0.031	0.568 **	0.577 **	0.310 **	0.314 **

Note: Q_Creat—creativity quotient, CTD_RO—critical thinking disposition reflective skepticism, CTD-CO—critical thinking disposition critical openness, GRIT, SkO—skeptical optimism, CSE—core self-evaluation, and SEOpt—self-efficacy optimism; factors 1 to 3 refer to the 17-item scale, while the same factors labeled SH refer to the short 9-item scale, * *p* < 0.05, ** *p* < 0.01.

**Table 7:** The results of the OLS regression analyses with all criterion variables and the subscales of skeptical optimism.

	Q_Creat	Self-Efficacy Optimism	GRIT Consistency	GRIT Perseverance	CSE	CTD_RO	CTD_CO
Constant	−0.49 (3.40)	1.19 (0.63)	1.01 (1.17)	1.88 (0.86)	3.13 *** (0.68)	1.32 * (0.68)	1.42 * (0.55)
Gender	0.12 (0.48)	−0.05 (0.09)	0.51 ** (0.16)	0.29 * (0.12)	0.00 (0.10)	0.34 *** (0.09)	0.05 (0.08)
Age	0.10 (0.13)	0.01 (0.02)	0.03 (0.05)	−0.01 (0.03)	0.00 (0.03)	0.02 (0.03)	0.02 (0.02)
Factor 1 (curiosity in mastering novel and challenging tasks)	0.85 * (0.34)	0.15 * (0.02)	−0.20 (0.12)	0.09 (0.09)	0.14 * (0.07)	0.12 (0.07)	0.21 *** (0.06)
Factor 2 (tenacity in scrutinizing difficult problems)	0.08 (0.35)	0.55 *** (0.06)	0.67 *** (0.12)	0.62 *** (0.09)	0.35 *** (0.07)	0.19 ** (0.07)	0.06 (0.06)
Factor 3 (inquisitiveness in addressing challenges)	0.41 (0.28)	−0.11 * (0.05)	−0.37 *** (0.10)	−0.31 *** (0.07)	−0.37 *** (0.06)	0.28 ** (0.06)	0.26 *** (0.05)
F	3.79 *	29.06 ***	10.36 ***	16.84 ***	14.97 ***	12.14 ***	20.26 ***
R^2^	0.09	0.44	0.22	0.31	0.28	0.24	0.35

Note: Q_Creat—creativity quotient, CTD_RO—critical thinking disposition reflective skepticism, CTD-CO—critical thinking disposition critical openness, CSE—core self-evaluation; * *p* < 0.05, ** *p* < 0.01, *** *p* < 0.001.

**Table 8:** The results of the OLS regression analyses with all criterion variables and the subscales of skeptical optimism (short version).

	Q_Creat	Self-Efficacy Optimism	GRIT Consistency	GRIT Perseverance	CSE	CTD_RO	CTD_CO
Constant	−0.42 (3.40)	1.42 (0.67)	1.33 (1.19)	2.43 (0.95)	3.38 (0.70)	1.44 * (0.698)	1.52 ** (0.57)
Gender	0.09 (0.48)	−0.04 (0.10)	0.53 ** (0.17)	0.29 * (0.13)	0.005 (0.10)	0.34 *** (0.10)	0.04 (0.08)
Age	0.11 (0.13)	0.02 (0.03)	0.03 (0.05)	−0.01 (0.04)	0.00 (0.03)	0.02 (0.03)	0.02 (0.02)
Factor 1SH (curiosity in mastering novel and challenging tasks)	0.79 ** (0.30)	0.14 * (0.06)	−0.22 * (0.11)	0.09 (0.08)	0.09 (0.06)	0.12 * (0.06)	0.19 *** (0.05)
Factor 2SH (tenacity in scrutinizing difficult problems problems)	0.17 (0.30)	0.44 *** (0.06)	0.54 *** (0.11)	0.38 *** (0.08)	0.30 *** (0.06)	0.14 * (0.06)	0.06 (0.05)
Factor 3SH (inquisitiveness in addressing challenges)	0.32 (0.26)	−0.08 (0.05)	−0.32 *** (0.09)	−0.24 ** (0.07)	−0.33 *** (0.05)	0.17 ** (0.05)	0.23 *** (0.04)
F	3.72 **	20.61 ***	8.63 ***	7.52 ***	12.11 ***	10.66 ***	17.40 ***
R^2^	0.09	0.35	0.19	0.17	0.24	0.22	0.32

Note: Q_Creat—creativity quotient, CTD_RO—critical thinking disposition reflective skepticism, CTD-CO—critical thinking disposition critical openness; * *p* < 0.05, ** *p* < 0.01, *** *p* < 0.001.

**Table 9:** Means, standard deviations, and correlations for variables in Study 2.

	Mean	SD	1	2	3	4	5	6	7	8	9	10	11
1. Gender	0.6523	0.47716	1										
2. Age	23.72	6.435	−0.036	1									
3. Educ_level	1.49	0.680	−0.092	0.414 **	1								
4. CAQ	6.8729	7.13305	0.081	−0.068	0.110 *	1							
5. Factor_1	3.8695	0.72190	−0.073	0.031	0.080	0.169 **	1						
6. Factor_2	3.8552	0.66818	−0.012	0.135 **	0.099 *	0.123 *	0.683 **	1					
7. Factor_3	3.8921	0.73547	0.033	0.019	−0.009	0.135 **	0.260 **	0.304 **	1				
8. SkO_Tot	3.8693	0.58637	−0.028	0.090	0.093	0.177 **	0.899 **	0.863 **	0.522 **	1			
9. Factor_1SH	3.9097	0.80204	−0.066	0.018	0.115 *	0.163 **	0.944 **	0.641 **	0.222 **	0.844 **	1		
10. Factor_2SH	3.8985	0.72984	0.008	0.134 **	0.094	0.125 *	0.660 **	0.933 **	0.319 **	0.822 **	0.610 **	1	
11. Factor_3SH	3.8122	0.82213	0.028	0.000	−0.024	0.101 *	0.189 **	0.250 **	0.965 **	0.450 **	0.148 **	0.268 **	1
12. SkO_TotShort	3.8734	0.58526	−0.013	0.064	0.081	0.174 **	0.794 **	0.798 **	0.686 **	0.938 **	0.780 **	0.820 **	0.647 **

Note: CAQ—creative achievement quotient, SkO—skeptical optimism; factors 1 to 3 refer to the 17-item scale, while the same factors labeled SH refer to the short, 9-item scale * *p* < 0.05, ** *p* < 0.01.

## Data Availability

The data analyzed in the current study are available from the corresponding author on motivated and reasonable request.

## Appendix A

**Table A1:** Initial item pool and their respective factor loading derived from EFA in Study 1 and correlations with criterion variables.

Item	Factor	EFA FL	Experts RWG	Creat Quot	CriticThink	CSE	GRITPers	GRIT Consis	SelfEffOptim
1. My persistence helps me deal with any difficult life situation	2	0.501	0.82	0.092	0.255 *	0.266 **	0.446 **	0.156 *	0.438 **
2. Curiosity makes me able to fulfill new and challenging tasks	1	0.575	0.82	0.268 **	0.353 **	0.259 **	0.294 **	0.103	0.417 **
3. For each problem I am confronted with I will find a solution because I tend to think a lot about things.	1	0.470	1	0.082	0.347 **	0.139	0.178 *	0.194 **	0.347 **
4. I always push through any problem I am faced with.	2	0.702	0.54	−0.041	0.380 **	0.386 **	0.508 **	0.415 **	0.486 **
5. I am excited when faced with new challenges.	2	0.700	1	0.093	0.220 *	0.238 **	0.285 **	0.118	0.457 **
6. I am willing to engage and persist in a complex task because I always find a way to get it done.	2	0.415	0.68	0.231 **	0.414 **	0.250 **	0.279 **	0.151 *	0.464 **
7. I believe I will always find a way when dealing with difficult challenges.	2	0.527	0.63	0.160 *	0.243 *	0.304 **	0.280 **	0.215 **	0.555 **
8. I find it difficult to believe there is only one solution to every problem			1	0.158 *	00.177	0.139	0.063	−0.039	0.165 *
9. I don’t believe in giving up when faced with a difficult situation.	2	0.563	0.47	0.109	0.264 **	0.246 **	0.271 **	0.177 *	0.348 **
10. I enjoy complexity in life due to my curiosity	1	0.624	0.77	0.240 **	0.366 **	0.074	0.034	−0.083	0.280 **
11. I have the drive to complete novel tasks no matter their complexity	2	0.635	0.82	0.086	0.331 **	0.252 **	0.467 **	0.269 **	0.491 **
12. I like exploring new and challenging tasks	1	0.773	1	0.220 **	0.377 **	0.187 **	0.249 **	0.027	0.412 **
13. I like experimenting because it challenges me to think quickly and flexibly	1	0.765	0.65	0.253 **	0.319 **	0.119	0.109	−0.066	0.300 **
14. I like to think out of the box when faced with difficult/new problems.	1	0.554	0.77	0.241 **	0.474 **	−0.020	−0.034	−0.131	0.190 **
15. I master difficult problems considering the situation as a whole			0.74	0.193 **	0.389 **	0.130	0.066	0.055	0.289 **
16. I persevere in face of difficulties due to my diligence in seeking relevant information	2	0.616	0.54	0.128	0.456 **	0.349 **	0.449 **	0.216 **	0.470 **
17. I see the life challenges as opportunities to grow			0.82	0.057	0.375 **	0.384 **	0.383 **	0.210 **	0.389 **
18. I welcome every new challenge because it triggers my inquisitiveness	1	0.685	0.73	0.134	0.432 **	0.278 **	0.293 **	0.090	0.413 **
19. My curiosity helps me deal with new and challenging tasks	1	0.693	1	0.209 **	0.444 **	0.181 *	0.205 **	−0.019	0.251 **
20. Once I have figured out a solution for a difficult problem, I don’t usually stop searching for the best solution			1	0.110	0.300 **	.122	0.138	0.086	0.304 **
21. There is no task that is too demanding for me			0.63	−0.026	0.252 *	0.229 **	0.327 **	0.215 **	0.521 **
22. For each problem I am confronted with, I wait to decide until I can get more information			1	0.039	0.226 *	−0.159 *	0.050	−0.259 **	−0.060
23. I dislike having to make important decisions in life without time to carefully reflect on alternatives			0.65	0.032	00.143	−0.147 *	−0.007	−0.076	−0.042
24. In novel tasks, I don’t accept other people’s explanations without further questioning			0.82	0.208 **	0.231 *	−0.059	−0.046	−0.059	0.155 *
25. In new situations, I usually evaluate the evidence even if it derived from valid testing			0.82	0.186 **	0.323 **	0.062	0.088	0.049	0.320 **
26. I enjoy the process of seeking additional information even when solutions are readily available			0.63	0.180 *	0.466 **	0.159 *	0.137	0.086	0.352 **
27. When first presented with a new problem, I tend not to take things at face value.			0.73	0.090	0.349 **	0.156 *	0.270 **	0.133	0.271 **
28. I often wonder about why something works or doesn’t work.	3	0.407	0.82	0.172 *	0.409 **	−0.119	−0.057	−0.151 *	0.078
29. In my life, I tend to question even most basic assumptions	3	0.462	1	0.095	0.416 **	−0.257 **	−0.172 *	−0.274 **	0.107
30. I sometimes overanalyze a problem because I want to make sure I reach the best solution.	3	0.798	1	0.123	0.379 **	−0.257 **	−0.038	−0.109	0.085
31. I sometimes overanalyze a new situation because I want to make sure I understand all aspects of it.	3	0.738	0.82	0.210 **	0.387 **	−0.137	0.028	−0.044	0.113

Note: * *p* < 0.05, ** *p* < 0.01.
